# Advancing Digital Workflows for Refractive Error Measurements

**DOI:** 10.3390/jcm9072205

**Published:** 2020-07-12

**Authors:** Arne Ohlendorf, Alexander Leube, Siegfried Wahl

**Affiliations:** 1 Institute for Ophthalmic Research, Center for Ophthalmology, Eberhard Karls University of Tuebingen, Elfriede-Aulhorn-Straße 7, 72076 Tuebingen, Germany; arne.ohlendorf@medizin.uni-tuebingen.de (A.O.); alexander.leube@uni-tuebingen.de (A.L.); 2 Technology & Innovation, Carl Zeiss Vision International GmbH, Turnstrasse 27, 73430 Aalen, Germany

**Keywords:** refractive error, visual acuity, myopia

## Abstract

Advancements in clinical measurement of refractive errors should lead to faster and more reliable measurements of such errors. The study investigated different aspects of advancements and the agreement of the spherocylindrical prescriptions obtained with an objective method of measurement (“Aberrometry” (AR)) and two methods of subjective refinements (“Wavefront Refraction” (WR) and “Standard Refraction” (StdR)). One hundred adults aged 20–78 years participated in the course of the study. Bland–Altman analysis of the right eye measurement of the spherocylindrical refractive error (M) identified mean differences (±95% limits of agreement) between the different types of measurements of +0.36 D (±0.76 D) for WR vs. AR (*t*-test: *p* < 0.001), +0.35 D (± 0.84 D) for StdR vs. AR (*t*-test: *p* < 0.001), and 0.0 D (± 0.65 D) for StdR vs. WR (*t*-test: *p* < 0.001). Monocular visual acuity was 0.0 logMAR in 96% of the tested eyes, when refractive errors were corrected with measurements from AR, indicating that only small differences between the different types of prescriptions are present.

## 1. Introduction

Subjective refraction is a key measure in the optometric and ophthalmic field. Typically, the combination of the most positive lenses (spherical and astigmatic) that provides the highest visual acuity is determined in this process that follows a predefined workflow [1]. Since this measurement is by its definition subjective, the repeatability and the precision of the measurement within the same and between different examiner can vary and was reported to have a 95% limit of agreement (LoA) between ±0.27 and ±0.75 D for the spherical equivalent refractive error [2,3,4,5]. To improve the repeatability of the technique and to make the process easier and more time efficient, it is common to use either objective data, which are obtained from an autorefractor, or the currently worn prescription as a starting point for the subjective assessment of individual refractive errors. So far, studies examining different objective autorefractors have shown that these are repeatable, under noncycloplegic as well as cycloplegic conditions. In addition, 95% limits of agreement (95% LoA) between ±0.35 and ±0.72 D for the measurement of the spherical refractive error under noncycloplegic conditions were reported [2,6,7]. Nowadays, aberrometers are used to describe the eyes aberrations in more detail. Although lower order aberrations affect vision most and account for approximately 90% of the overall aberrations, [8] also higher order aberrations such as spherical aberration, coma, and trefoil can significantly degrade the quality of the optical image received by the retina [9]. Limits of agreement for the wavefront measurement of the spherical error were found to be between ±0.2 (for a pupil diameter of 4.0 and 6.7 mm) [10] and ±0.55 D [11] (for pupil diameters of 4.0 and 6.0 mm) and did not differ between several measurements, also without the use of an cycloplegic agent to block accommodation [11]. Additionally, e.g., to consider different lighting conditions [12], wavefront sensors allow to compute the refractive errors for different pupil sizes and/or allow the consideration of different higher order aberrations [13].

The purpose of this study was to evaluate the agreement between three different methods to assess the spherocylindrical correction. Method 1 represents an Autorefraction (AR) approach using aberrometry measurements; in Method 2, the sphere of the refractive correction of Method 1 was adjusted; and in Method 3, the sphere, the cylinder, and its axis of the prescription from Method 1 were adjusted. The study investigates the question how much refinement a clinician has to perform subjectively so that the final prescription results in a good and acceptable visual acuity and therefore, saves time.

## 2. Experimental Section

### 2.1. Subjects

Correction of spherocylindrical errors was measured in 100 participants, aged 20–78 years (mean: 38.7 ± 13.2 years). Participants had a mean spherical refractive error (computed from the objective wavefront measurement for a 3 mm pupil diameter and a 12 mm vertex distance) of −1.40 ± 2.15 D (−12.75 to +2.5 D) and a range of astigmatic refractive errors between 0 and −3.25 D. Inclusion criterions for participation was (a) refractive error of less than ±13.00 D sphere, ≤ −6.00 D of astigmatism, and (b) best corrected visual acuity of minimum 0.1 logMAR (logarithm of the minimum angle of resolution). Participants with known/reported ocular diseases were not allowed to participate in the course of the study. The study followed the tenets of the Declaration of Helsinki and was approved by the Institutional Review Board of the Medical Faculty of the University of Tubingen (392/2015BO2). Informed consent was obtained from all participants after the content and possible consequences of the study had been explained.

### 2.2. Experimental Procedures to Measure Refractive Errors Objectively and Subjectively

Objective measurement of the refractive error of each eye was obtained once prior to the subjective measurements by author A.O. using a wavefront aberrometer (i.Profiler plus, Carl Zeiss Vision GmbH, Aalen, Germany). Refractive correction (Sphere, Cylinder, Axis) was calculated from the objectively measured wavefront errors using the lower order Zernike polynomials for a pupil diameter of 3 mm and a vertex distance of 12 mm [14]. Using the lower order terms for the spherical, straight, and oblique astigmatic component of the wavefront shapes, a simple sphere fitting was performed to calculate the best correction [13], based on the purely objective measurements without using higher order terms.

One examiner (author A.L.) subjectively measured the refractive errors using a digital phoropter (ZEISS VISUPHOR 500, Carl Zeiss Vision GmbH, Aalen, Germany) and the two methods “Wavefront Refraction” and “Standard Refraction.” The digital phoropter was operated via a tablet PC (iPad Air, Apple, Cupertino, CA, USA). The screen of the tablet PC was covered with black paper in the area where refractive readings were displayed to exclude any influence or bias on the examiner, during the assessment of the spherocylindrical prescriptions with both subjective methods. The sequence of testing of the “Wavefront Refraction” and “Standard Refraction” was randomized between each participant, but the refraction always started with the right eye. As the ZEISS VISUSCREEN 500 also registers the time that is needed for the binocular measurement of the refractive error, time was recorded and analyzed. During the “Wavefront Refraction,” only the spherical error of the refractive error was adjusted, whereas during the “Standard Refraction,” the spherical error as well as the astigmatism was adjusted. For both methods, the used optotypes (letters) were displayed on an LCD screen (ZEISS VISUSCREEN 500, Carl Zeiss Vision GmbH, Germany) at 6 m distance and followed the EDTRS-layout. Lighting conditions were in the range of 250 cd/m^2^ and followed the international standard ISO 8596:2018 [15]. In the case of the assessment of the spherical error under monocular conditions, three lines of letters (each consisting of five single optotypes) were presented on the screen, and the lowest presented acuity was 0.1 logMAR. In case astigmatism was adjusted, also letters were used, but only one line with five letters was presented on the screen, and the letter size was 0.1 logMAR steps bigger than the smallest optotypes visible after correction of the spherical error. A simultaneous cross-cylinder was used to measure the astigmatism and its axis. After both procedures, the dissociated binocular balance was tested, using a single line of letters that was dissociated using a prism (power: right eye 3 D base up, left eye 3 D base down). The size of the presented letters was set to 0.3 logMAR and spherical blur of +0.5 D was added to both eyes. Then, additional plus lenses were induced until the participants perceived the images in both eyes equally blurred. Afterwards, to achieve a binocular balance as endpoint for refraction, the nondissociated binocular best corrected vision was tested using letters, presented in an ETDRS layout and while the blurring lenses from the previous test were removed. The prescription was changed until visual acuity was maximal in both eyes.

### 2.3. Assessment of Visual Acuity and Subjective Preference

Visual acuity for each eye was assessed after correction of the refractive errors with each of the earlier described methods. As stated above, each acuity line consisted of five optotypes and each correctly identified single letter was scored 0.02 logMAR units [16]. Highest visual acuity describes the smallest optotype for the given test distance that was correctly identified. At the end of the experiment, and in order to not only rely on the subjective prescription determined by an optometrist that corrects the refractive error, the participants were asked, which of the three corrections they preferred most. This was done using the capability of the digital phoropter in conjunction with the tablet PC to store the refractive readings for each single participant and to show these in a fast sequence at the end of an examination. During such comparison, the subjects viewed a line of 5 optotypes with a size of 0.1 logMAR, presented on the described LCD display at the test distance of 6 m. Using this possibility, the subject was able to compare the different corrections that were obtained with one of the three methods. The following direct comparisons were evaluated in a randomized order: (a) Autorefraction vs. Wavefront Refraction, (b) Autorefraction vs. Standard Refraction, (c) Wavefront Refraction vs. Standard Refraction, and (d) Autorefraction vs. Wavefront Refraction vs. Standard Refraction.

### 2.4. Analysis and Statistics

The right eye of each subject was used for the analysis of the data. Refractive measurements were analyzed for the spherical refractive error (S), the spherical equivalent refractive error (M), and the cross-cylinder components J0 and J45 that were introduced by Thibos, Wheeler, and Horner [17]. The agreement between the different methods for the spherocylindrical correction of the right as well as for the visual acuity was tested using Bland–Altman analysis [18]. The calculated mean difference between two methods represents the estimated bias of one method, and the standard deviation of the differences measures the random fluctuations around this mean. The computation of the 95% limit of agreement (calculated as 1.96*standard deviation) describes how far apart measurements by two methods were more likely to be for most individuals. Statistical analyses were performed with the statistics software package JMP 11.0 (SAS Institute, Cary, USA). In case of the Bland–Altman analysis, a *t*-test was performed to calculate statistical power between the compared methods. A one-way ANOVA with post hoc Tukey HSD test was used to test whether the used method of refraction had an influence on the measurement of the spherical equivalent refractive error. A *t*-test was used to compare the time that each of the subjective method needed to assess the spherocylindrical refraction.

## 3. Results

### 3.1. Agreement Between Subjective and Objective Refractions

**Figure 1:**
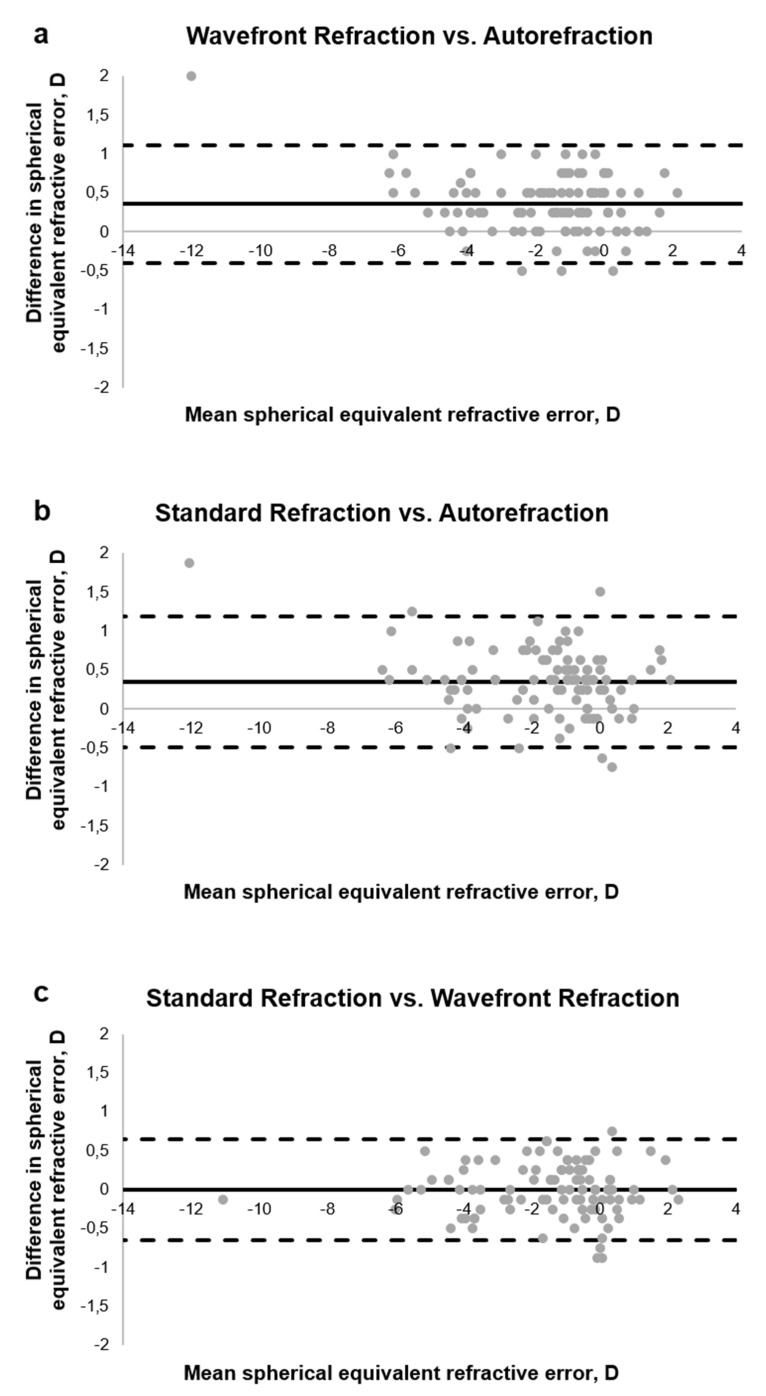
Bland–Altman plot for the comparison of the spherical equivalent refractive error after binocular testing with (**a**) Wavefront Refraction vs. Autorefraction, (**b**) Standard Refraction vs. Autorefraction, and (**c**) Standard Refraction vs. Wavefront Refraction.

Figure 1a represents the Bland–Altman plots for the comparison of the spherical equivalent refractive error computed from Autorefraction vs. the Wavefront Refraction and Figure 1b represents the comparison between the Autorefraction vs. the Standard Refraction.

When comparing the Wavefront Refraction as well as the Standard Refraction to the objective data obtained with the aberrometer, the calculated bias was around 0.3 D, resulting in too negative readings of the spherical errors when using the aberrometer (for details, see Table 1). The limits of agreement are smaller, in case Autorefraction was compared to the Wavefront Refraction, and this result is attributed to the fact that only the spherical refractive error was adjusted between both methods. In addition, when the cylinder and its axis were rechecked (comparison between Autorefraction and Standard Refraction, Figure 1b), the limits of agreement slightly increased because the power of the cylindrical error may have changed between both methods. The one-way ANOVA with post hoc Tukey HSD test revealed that the used method (F4,13 = 0.89, *p* = 0.4) did not influenced the measured spherical equivalent error. Table 1 summarizes the mean differences as well as the 95% limits of agreement for all three possible comparisons as well as for the spherical refractive error (S) and the three power vectors M, J0 and J45.

Comparisons of the power vectors J0 and J45 showed very small mean differences and low 95% limits of agreement, when methods were compared with each other. Since in case of the refractive measurement that was based on the objective Autorefraction and the Wavefront Refraction, the astigmatism and its axis were not changed, the descriptive as well as statistical analysis was not assessed. For the spherical and spherical equivalent refractive error, the statistical analysis revealed a significant difference between the measurements obtained with the objective measurement calculated from the wavefront errors and both subjective refractive measurements (Wavefront Refraction and Standard Refraction).

### 3.2. Differences in Cylinder and Its Axis

**Figure 2:**
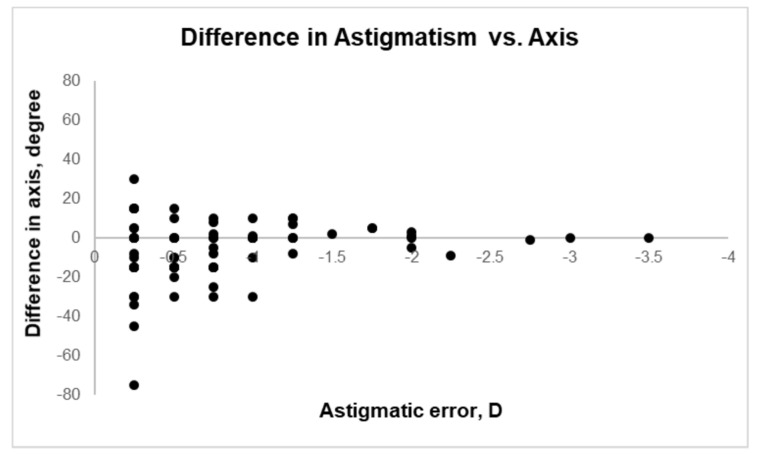
Difference of the cylinder-axis (deg) between Standard Refraction and Autorefraction as a function of the power of astigmatic error (D).

The bias between two methods as well as the 95% limit of agreement were calculated for the power vectors M, J0 and J45. These power vectors lend itself to calculations of sums, differences, and averages and are the correct form for describing refractions for such measures. Nevertheless, especially in case of the cylinder and its axis, these are not the ideal notations to get an overview, if the data obtained with an autorefractor and with a subjective refraction are equal or differ, e.g., depending on the amount of astigmatism or on its axis. Therefore, a comparison between the measured astigmatism and its axis between the autorefraction data and the values from the Standard Refraction was performed, following the analysis by Grein et al. [19]. In Figure 2, the discrepancy for the axis, based on the objective measurement of the wavefront errors and with the Standard Refraction, is analyzed depending on the power of the astigmatic error. It must be noted that in this analysis, an astigmatism was only considered if it was found in both methods of refraction, therefore, only data of 85 eyes were analyzed. A positive difference means a change of the axis into the clockwise direction, whereas a negative difference is equal to a counterclockwise change.

From Figure 2 it can be concluded that astigmatic errors of ≤ −1.0 D were very frequent (79% of the 85 right eyes) and that the difference between the objective axis of the correcting cylinder and the subjective axis differed especially at very small amounts of astigmatism. Vice versa, the difference between the axis decreased with increasing power of astigmatism, indicating that the quality of the objectively measured wavefront error for the cylinder axis increased with increasing power of astigmatism. To investigate, if a measured astigmatism needs to be corrected, the difference between the cylindrical error of the right eye when measured with the Standard Refraction and the objective measurement of the wavefront error was analyzed. The results gave evidence that the power of the astigmatic error based on the objective measurement of the wavefront error was more negative when compared to the Standard Refraction, however, 74% of the measurements had a difference within ± 0.25 D.

### 3.3. Visual Acuity from Different Correction Methods

The monocular visual acuity that was achieved with either one of the procedures was analyzed, as one can predict that a spherical refractive error of 0.25 D reduces the monocular visual acuity by 1 line (or 0.1 logMAR unit) [20,21], the achieved visual acuity (especially under monocular conditions) is a good indicator for (a) the quality of a method to detect a refractive error and (b) for the agreement between different methods. The analysis revealed that already 96% of the eyes had a visual acuity equivalent of 0.0 logMAR or better with correction data (sphere, cylinder, and axis) obtained from the aberrometer without further adjustments. This was slightly increased to 98%, when the refractive error was subjectively fine-tuned by an experienced optometrist (Wavefront Refraction, *n* = 98 eyes; Standard Refraction, *n* = 98 eyes). Table 2 summarizes the mean differences in visual acuity and the lower as well as upper limits for the single comparisons.

### 3.4. Subjective Preferences in Correction

**Figure 3:**
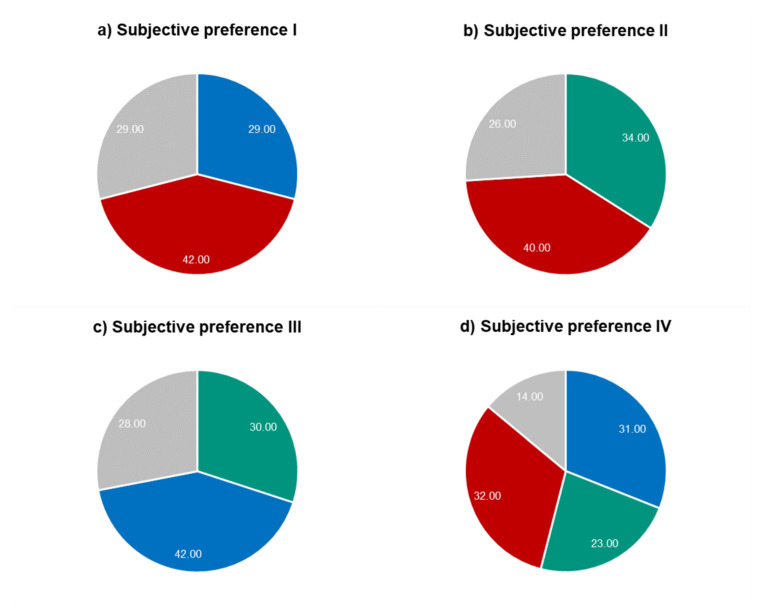
Subjective preferences for direct comparisons of methods (Figure 3a–c) and between methods. (**a**) Autorefraction (red) vs. Wavefront Refraction (blue), (**b**) Autorefraction (red) vs. Standard Refraction (green), (**c**) Standard Refraction (green) vs. Wavefront Refraction (blue), and (**d**) Standard Refraction (blue) vs. Wavefront Refraction (green) vs. Autorefraction (red). Gray area: no difference.

Figure 3 summarizes the findings of the subjective preference comparison, while the participants always had three possible decisions: whether they liked one of the corrections better than the other(s) or they were not able to see a difference.

When comparing the individual as well as averaged preferences, it becomes clear that participants preferred the correction based on objective measurement of the wavefront error most (Figure 3a,b). In case the Standard Refraction was compared to the Wavefront Refraction, subjects preferred the Wavefront Refraction more (42%, Figure 3c). When the participants were asked to compare all three methods with each other, the Autorefraction and Wavefront Refraction were rated similar (Autorefraction: 32% and Wavefront Refraction: 31%) and both ratings were higher compared to the Standard Refraction (23%).

### 3.5. Number of Decisions and Time for the Assessment of the Refraction

One main advantage of the Wavefront Refraction is that the number of decisions a patient has to make during the assessment of his or her refractive correction is reduced due to the fact that only the sphere under monocular as well as binocular conditions is rechecked by the eye care professional. Therefore, the number of decisions in each test was recorded and analyzed. During the process of the Wavefront Refraction, participants had to take 17 decisions on average, while the number of decisions increased to 25 in case of the Standard Refraction—an increase of roughly 50%. Since in the case of the Wavefront Refraction, the power as well as the axis of an existing astigmatism was not tested, one can assume that this procedure is much faster compared to the Standard Refraction. In case, the average time that was needed for the Wavefront Refraction was 353 ± 82 s (range 214–575 s), while this increased to 539 ± 119 s (range: 306–937 s) in case of the Standard Refraction (*t*-test: *p* < 0.001).

## 4. Discussion

Conventional autorefractors and subjective refraction following a standardized protocol are currently the gold standard in the assessment of refractive errors. Aberrometers have gained attention in optometric as well as ophthalmological settings since they are able to measure lower and higher order aberrations. The study has assessed the accuracy of Autorefraction while comparing the refractive correction as well as the visual acuity with two subjective refinement methods.

### 4.1. Agreement and Percentage of Agreement Between Subjective and Objective Refractions

Aberrometers have been shown to be reasonable, accurate, and repeatable [22,23,24]. Several investigations [25,26] have compared the spherical-equivalent refractive error of autorefraction to subjective refraction and the observed difference was less than or equal to ±0.50 D between 70% and 74% of the time. In case of the i.Profiler plus from ZEISS, the percentages of agreement for differences in the spherical refractive error of ±0.50 D were higher and calculated to be minimum 76%, when Standard Refraction was compared to the Autorefraction. One possible explanation is given by the internal fixation target, which is additionally blurred optically during the measurement, to avoid instrument myopia. Nevertheless, both subjective methods had fewer negative values in the final spherical equivalent refractive error compared to the objective method, resulting in a mean difference for the sphere of 0.36 D for Autorefraction vs. Wavefront Refraction and 0.35 D for Autorefraction vs. Standard Refraction. Lebow and Campbell (2014) investigated differences of the objective measure from the ZEISS i.Profiler plus to a conventional subjective measurement of the spherical equivalent refractive error in adults and reported a mean difference of 0.11 D (more negative readings with the i.Profiler plus) [25]. As the one-way ANOVA with post hoc Tukey HSD test showed that the device had no effect on the measured spherical equivalent error, one cannot assume that a systematic bias exists between the used methods and different explanations can account for the observed differences. First, subjects might have accommodated during the objective refraction, but there was only a small trend that younger participants showed higher differences than older participants (R = −0.2, *p* = 0.06). Second, and as already described by others, roughly 0.25 D of the observed difference could also be accounted to the fact that the location of infrared scatter layer is different than photoreceptor layer [27]. Third, chromatic aberration might play a role, but researchers have not found an improvement in accuracy between objective Autorefraction from wavefront errors and subjective refraction, in case polychromatic metrics were used to compute refractive correction or by taking into account the Stiles–Crawford effect [28].

### 4.2. Differences in Cylinder and Its Axis

When comparing the axis of the astigmatic error that was obtained with the aberrometer to the Standard Refraction, the gained results could lead to the interpretation that objective measurements are not very precise. From investigations of the interexaminer agreement of the axis of a correcting cylindrical lens, the same distribution of difference is known; therefore, it can be concluded that such differences can occur not only between an objective as well as a subjective method but also when different examiner measure the refractive errors of an individual. [18] For the difference between the power of a correcting lens, assessed objectively and subjectively, a mean difference of 0.12 D was observed, with 95% limits of agreement of ± 0.66 D. Lebow and Campbell, who also used the Autorefraction data from the ZEISS i.Profiler plus, compared these data to their Standard Refraction and the observed that mean differences are comparable to the current study (mean difference 0.02 D) [25]. In an investigation by Wosik et al. showed that the assessment of the astigmatic error is superior using aberrometer devices compared to standard autorefractors [29].

### 4.3. Impact of Higher Order Aberrations on Refraction Assessment

**Figure 4:**
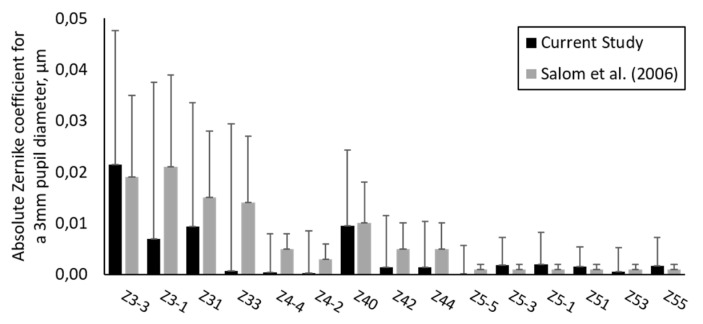
Comparison of the absolute Zernike coefficient (µm) for the higher order aberrations until fifth order from the current study to normal human subjects for a 3 mm pupil diameter (data from Salmon et al. [33]).

Additionally to the lower order aberrations, used to calculate the sphere, cylinder and axis, aberrometry provides information about higher order aberrations (HOAs) like coma, trefoil, or spherical aberration. Previous studies showed that the usage of HOAs can improve precision in interexaminer evaluation [13], especially in highly aberrated eyes, like keratoconic eyes [30,31]. However, image quality-based predictions in normal eyes range within the here reported limits of agreements towards the subjective refraction [32]. Since the current investigation enrolled only normally sighted participants, the amount of HOAs in the cohort is low and comparable to already reported values [33], as shown in Figure 4. Furthermore, the objective aberrometry measurements that were evaluated for a pupil diameter of 3 mm reduce the impact even further.

### 4.4. Visual Acuity with Each Correction

Visual acuity of 0.0 logMAR was achieved in 96% of the tested right eyes with the spherocylindrical prescriptions from the aberrometer. This portion increased to 98% after balancing the spherical error, in case of the Wavefront Refraction as well as in case of the Standard Refraction, where sphere, cylinder, and axis were balanced. This small increase in visual acuity can be explained by the fact that the spherical refractive error was around 0.3 D too negative with the objective method. The fact that visual acuity increased only slightly, when the refractive error was adjusted with both of the two subjective methods, is a good indicator for the fact that autorefraction measurement of spherocylindrical corrections are very reliable compared to two subjective methods that were evaluated.

### 4.5. Subjective Preferences in Correction

On average, participants preferred spherocylindrical correction from the autorefractor most, when compared to both subjective methods (Wavefront Refraction as well as Standard Refraction). This result can be explained by the fact that, especially, the measurements of the sphere were slightly more myopic with the autorefractor compared to both mentioned methods. This shift towards more negative power would result in a slightly higher contrast of the image on the retina that especially myopes prefer more compared to blur that would have been the result if spherical corrections were more positive. Nevertheless, since these comparisons were done with optotypes that had a size of 0.1 logMAR, one has to be careful with the interpretation of the results. In the future, it would be better to individually test this comparison at the threshold of the acuity that the single participant is able to read (in the case one wants to use optotypes) or to present more natural stimuli during the process of comparison, since optotypes are quite unnatural and the prescription is needed for the daily life.

### 4.6. Number of Decisions and Time for the Assessment of the Refraction

On average, 50% less decisions had to be made by each participant when refraction was measured using the Wavefront Refraction compared to the Standard Refraction. This small amount of decisions indicates that the prescriptions from the aberrometer are very close to the final value, when, e.g., compared to the refractive correction obtained with a Standard Refraction. Additionally, Wavefront Refraction was 50% faster than the Standard Refraction. Since the results from the wavefront-based subjective assessment of the refractive errors were shown to be comparable to the standard procedure in the measurement of refractive errors, one can conclude that the combination of the Autorefraction and Wavefront Refraction will result in acceptable prescription for patient in a fast manner.

## 5. Conclusions

Aberrometry may change the way refractive errors are measured clinically and provides a tremendous amount of data about the aberrations of the eye. Spherocylindrical prescriptions that were obtained by measuring wavefront errors, provided reliable information for the further correction of lower order aberrations with a subjective method. Autorefraction data was slightly more negative for spherical as well as astigmatic errors, but > 70% of the spherical equivalent error were within ±0.5 D compared to the conventional subjective refraction. The combination of the Autorefraction and Wavefront Refraction, while refining only the spherical refractive error, will result in acceptable prescription for the patient and can significantly save time in the assessment of the refraction.

## Figures and Tables

**Table 1:** Descriptive and statistical analysis for the comparison of the three methods to assess the habitual refractive errors.

		Mean Difference, D	95% Limit of Agreement, D	*p*
Wavefront Refraction vs. Autorefraction	S *	+0.36	±0.76	< 0.001
	M’	+0.36	±0.76	< 0.001
	J0°	Not assessed		
	J45^	Not assessed		
Standard Refraction vs. Autorefraction	S *	+0.27	±0.88	< 0.001
	M’	+0.35	±0.84	< 0.001
	J0°	−0.05	±0.35	< 0.001
	J45^	+0.02	±0.27	< 0.01
Standard Refraction vs. Wavefront Refraction	S *	0.09	±0.65	= 0.6
	M’	+0.0	±0.65	= 0.3
	J0°	-0.05	±0.36	< 0.001
	J45^	+0.02	±0.27	< 0.01

S * = spherical refractive error; M’ = spherical equivalent refractive error; J0° = Jackson cross cylinder at 0°; J45^ = Jackson cross cylinder at 45°.

**Table 2:** Mean differences in visual acuity reached with each method and 95% limits of agreements.

	Mean Difference in VA, logMAR	95% Limit of Agreement, logMAR	*p*
Wavefront Refraction vs. Autorefraction	−0.02	±0.07	< 0.001
Standard Refraction vs. Autorefraction	−0.03	±0.10	< 0.001
Standard Refraction vs. Wavefront Refraction	−0.01	±0.09	= 0.02
